# Serum 25-Hydroxyvitamin D and the Incidence of Acute Viral Respiratory Tract Infections in Healthy Adults

**DOI:** 10.1371/journal.pone.0011088

**Published:** 2010-06-14

**Authors:** James R. Sabetta, Paolo DePetrillo, Ralph J. Cipriani, Joanne Smardin, Lillian A. Burns, Marie L. Landry

**Affiliations:** 1 Department of Medicine, Yale University School of Medicine, New Haven, Connecticut, United States of America; 2 Section of Infectious Diseases, Greenwich Hospital, Greenwich, Connecticut, United States of America; 3 Department of Physiology and Pharmacology, Wake Forest University School of Medicine, Winston-Salem, North Carolina, United States of America; 4 Department of Laboratory Medicine, Yale University School of Medicine, New Haven, Connecticut, United States of America; National Institute for Infectious Diseases L. Spallanzani, Italy

## Abstract

**Background:**

Declining serum concentrations of 25-hydroxyvitamin D seen in the fall and winter as distance increases from the equator may be a factor in the seasonal increased prevalence of influenza and other viral infections. This study was done to determine if serum 25-hydroxyvitamin D concentrations correlated with the incidence of acute viral respiratory tract infections.

**Methodology/Findings:**

In this prospective cohort study serial monthly concentrations of 25-hydroxyvitamin D were measured over the fall and winter 2009–2010 in 198 healthy adults, blinded to the nature of the substance being measured. The participants were evaluated for the development of any acute respiratory tract infections by investigators blinded to the 25-hydroxyvitamin D concentrations. The incidence of infection in participants with different concentrations of vitamin D was determined. One hundred ninety-five (98.5%) of the enrolled participants completed the study. Light skin pigmentation, lean body mass, and supplementation with vitamin D were found to correlate with higher concentrations of 25-hydroxyvitamin D. Concentrations of 38 ng/ml or more were associated with a significant (p<0.0001) two-fold reduction in the risk of developing acute respiratory tract infections and with a marked reduction in the percentages of days ill.

**Conclusions/Significance:**

Maintenance of a 25-hydroxyvitamin D serum concentration of 38 ng/ml or higher should significantly reduce the incidence of acute viral respiratory tract infections and the burden of illness caused thereby, at least during the fall and winter in temperate zones. The findings of the present study provide direction for and call for future interventional studies examining the efficacy of vitamin D supplementation in reducing the incidence and severity of specific viral infections, including influenza, in the general population and in subpopulations with lower 25-hydroxyvitamin D concentrations, such as pregnant women, dark skinned individuals, and the obese.

## Introduction

There are seasonal variations in the incidences of viral respiratory tract infections, such as those caused by influenza [1], respiratory syncytial virus [2], and rhinovirus [3]. Explanations for the seasonality of infections have considered the effects of environmental factors on the survival and transmission of the pathogens [4], [5] as well as on the behavior and susceptibility of the hosts [6].

Indoor crowding is commonly thought to contribute to the influenza epidemics seen each winter in temperate zones. However, influenza epidemics do not occur in the summer in crowded workplaces or other gatherings, despite the presence of the virus and a multitude of nonimmune persons [6], [7]. Moreover, influenza epidemics occur simultaneously at the same latitudes across the globe; this was the case even in times when transportation methods did not allow contact between persons over many degrees of longitude over a period of a few weeks [8].

There are well-documented seasonal variations in 25-hydroxyvitamin D (vitamin D) concentrations [9]-[11] and documented correlations between those concentrations and latitudes of residence [12], [13].

Vitamin D has known effects on the immune system. The production of the antimicrobial peptides cathelicidin by macrophages and β-defensin by endothelial cells is upregulated by vitamin D [14], [15]. These peptides may be involved in the direct inactivation of viruses [16], [17]. Vitamin D may modulate the production of cytokines, suppressing inflammation [18], and, thereby, reduce the severity of viral pneumonia [19].

The association between vitamin D deficiency and susceptibility to infections of the respiratory tract has been suggested for many years, but has not been definitively proven. Children with nutritional rickets developed rachitic lung due to infections of the respiratory tract [20], [21]. Cod liver oil, rich in vitamin D, was used to treat tuberculosis [22]. Epidemiologic studies have suggested a correlation between vitamin D concentrations and the incidence of respiratory infections [23], [24], including influenza [25].

There have been no prospective, observational studies following adult women and men with known vitamin D concentrations for the development of acute viral respiratory tract infections. Since it is unknown if an acute infection could transiently alter the 25-hydroxyvitamin D concentration and concentrations would be expected to decline over the fall and winter as latitude increases from the equator, serial serum concentrations would have to be obtained in a prospective study done in a temperate zone.

This study was undertaken to determine if there is any correlation between the incidence of acute viral respiratory tract infections and serum vitamin D concentrations as measured monthly from September 20, 2009 to January 10, 2010 in healthy adults living and/or working in or near Greenwich, CT, USA (latitude 41.038N, longitude 73.614W).

## Methods

### Participants

All participants signed informed consent approved by the Greenwich Hospital Institutional Review Board. Healthy adults, contacted through formal and informal presentations, were asked to volunteer to participate in this study. Exclusion criteria were any chronic pulmonary, cardiac, renal, hepatic, hematologic, neurologic, neuromuscular, or metabolic disorders (including diabetes mellitus); immunosuppression; pregnancy; and/or high dose aspirin therapy. Participants were not excluded if on thyroid or estrogen replacement therapy, oral contraceptives, or if they had mild seasonal allergies.

Participants agreed to donate one tube (7.5 cc) of blood monthly, starting in the third week of September 2009, for 4–5 months, depending on an interim analysis after the third blood draw. The participants were unaware of the nature of the substance being measured; they also understood they would be told the substance and their concentrations at the end of the study and that this data might be of benefit to them for health reasons unrelated to the study. The participants were asked to report any evidence of an acute respiratory tract infection (rhinorrhea, sore throat, and/or cough with or without fever, chills, myalgias, arthralgias), in which case they would be evaluated without charge at the study site.

### Determination of 25-hydroxyvitamin D concentrations

After obtained, sera were refrigerated and assayed within 4–48 hours in the Clinical Laboratory of Greenwich Hospital. Concentrations of 25-hydroxyvitamin D were measured in duplicate by a chemiluminescence immunoassay (Liaison®) [26]. The first concentrations were drawn from September 20–28, 2009, and then monthly thereafter. The investigators on site were blinded to the coded results, which were sent to the offsite investigator (PDP) who was not involved in clinical assessment.

### Clinical evaluation

Participants reporting any symptoms were seen the same day at the study site by one of the two board-certified Infectious Diseases investigators (JRS, RJC). Participants were interviewed, examined, and, if felt to have an acute respiratory tract infection, had a nasopharyngeal swab obtained for virology studies and bacterial cultures if a bacterial infection was suspected. The participants kept a diary of symptoms and were called every 1–3 days during the illness to review any signs or symptoms until asymptomatic. The duration of each symptom, the total illness duration, and any antimicrobials administered were recorded in the case report forms.

Between each monthly visit for 25-hydroxyvitamin D determinations, all of the participants were reminded every 10 days to report any illness. At each visit after the first, a clinical assessment was made by both of the Infectious Diseases investigators to determine if an illness being retrospectively reported by a participant appeared to have been an acute viral respiratory tract infection. In that case, an illness diary was completed, but no virology studies performed. At each monthly visit records were made for each participant regarding medications, herbals, supplements, vitamins, receipt of seasonal and/or 2009 H1N1 vaccine. At the first visit, the skin pigmentation of each participant was determined to be light (white-yellow), intermediate (tan-light brown), or dark (brown-black); and, the following were recorded: age, sex, height, weight, occupation, and contact information.

All data was entered into a secure web-accessible on-line database (MARVI) as well as into paper case report forms for back-up and quality verification purposes.

### Virology studies

All participants presenting with acute symptoms of a respiratory tract infection had a nasopharyngeal swab obtained and placed in viral transport media (M4, Remel, Lenexa, KS). These specimens were tested within 24 hours of collection at the Clinical Virology Laboratory at the Yale New Haven Hospital by cytospin-enhanced direct immunofluorescence (DFA) for adenovirus, parainfluenza types 1,2,3, respiratory syncytial virus, and influenza A and B (SimulFlour Respiratory Virus Screen, Light Diagnostics, Temecula, CA) [27]–[29]. At the end of the study, DFA-negative specimens that had been frozen at −70°C were assayed by RT-PCR for the predominant viruses known to be circulating during the study period and either not included in the DFA panel (rhinovirus and human metapneumovirus) or suboptomally detected (2009 H1N1 influenza) [30]–[32].

### Classification of illness

All ill participants were determined to either have a bacterial infection (e.g., streptococcal pharyngitis) or an acute viral respiratory tract infection, the latter of which were subclassified as either (1) an afebrile viral respiratory tract infection; (2) an influenza-like illness (ILI) with temperature >100°F, cough and/or sore throat in absence of a known cause; or, (3) a laboratory-confirmed viral infection.

Bacterial infections were not evaluated in this study and were excluded from the analysis.

### Statistical Analyses

Assuming an overall incidence of viral respiratory tract infections of 0.15 for the population over the time of study and a laboratory measurement coefficient of variation of 10% for serum vitamin D called for an enrollment of at least168 participants to achieve a Type I error of 0.05 and a Type II error of 0.2 if the odds ratio between the two groups for an event was ≥0.5 [33].

First, the initial serum 25-hydroxyvitamin D concentrations were examined with respect to the onset of respiratory tract viral infections seen over the entire study period. A non-linear pharmacodynamic concentration-response model was constructed, with parameters estimated by minimizing the log likelihood. Several families of sigmoid concentration-response model structures relating serum 25-hydroxyvitamin D concentration to the length of time of illness-free survival were evaluated, including exponential, extreme value, log-logistic, log-normal, normal, and Weibull functions.

Next, for each of the three observation periods a mean concentration of 25-hydroxyvitamin D was calculated for each participant by averaging the concentrations for that participant from before and after the observation period. Mean concentrations were used rather than concentrations extrapolated to the date of illness, as (1) it could not be assumed that the change in a concentration from the beginning to the end of an observation period was necessarily linear, and (2) concentrations had to be assigned to the participants who did not become ill.

Participants were stratified into two groups by presence or absence of an infectious event. An automated partition analysis [34] to determine what, if any, threshold of serum 25-hydroxyvitamin D would best discriminate between the two groups was undertaken for each period of observation and for the whole study. Parameters obtained from the partition analysis were compared to those obtained from the initial concentration-response curve in preparation for a subsequent survival analysis done to determine the magnitude of risk reduction.

For the survival analysis, participants who experienced an event (became infected) were assigned the mean vitamin D concentration for the period in which the event occurred. Participants who did not experience an event were assigned the minimum mean concentration taken over the three observation periods. This approach biased the study towards accepting the null hypothesis of no difference in event rate between the two groups.

A semi-parametric Cox proportional hazards model [35] was used to determine if participants stratified by high or low vitamin D concentrations were less likely to develop an acute viral respiratory tract infection. Participants who did not complete the study or who did not experience an event were right-censored to the time the study ended. Ties for time to event were handled using the Breslow likelihood. The regression parameters for the Cox model were estimated by the maximum likelihood method using JMP 5.0 (SAS, Cary, NC, USA).

The two groups were then examined in an analysis for differences in sex, age, skin pigmentation, use of herbals and supplements, use of vitamins other than D, use of vitamin D supplementation, and receipt of seasonal and 2009 H1N1 influenza vaccines prior to the observation periods. This analysis involved construction of a non-linear model relating skin pigmentation (ordinal scale 2 = light, 1 =  intermediate, 0 =  dark), gender (nominal F/M), age (yrs), height (in), weight (lbs), and vitamin D daily dose (IU) to the observed serum vitamin D concentration obtained at the first blood draw. The preferred model was chosen based on the explanatory vector of covariates achieving the lowest Akaike Information Criterion [36].

For each period of observation and for the study duration the incidences of viral respiratory infections were determined for participants with 25-hydroxyvitamin D concentrations above and below the partition value. Comparisons were made, and 95% confidence intervals for the mean and p values were calculated using a bootstrap resampling routine (over 100,000 trials) in MATLAB (The Mathworks, Natick, MA, US) with the resampling functions package from Resampling Statistics (Resampling Stats, Inc. Arlington, VA, USA). Observations were then made regarding the burden of illness by comparing days of viral respiratory illness per days of observation for participants with 25-hydroxyvitamin D concentrations above and below the partition value.

Lastly, the two groups were examined for differences in the number of participants who developed viral infections associated with positive virology laboratory studies.

## Results

One hundred ninety-eight participants, 85 men and 113 women, with an age range of 20–88, were enrolled in the study. Skin pigmentation was light (white-yellow) in 154, intermediate (tan-light brown) in 32, and dark (brown-black) in 12. One participant withdrew from the study days after the first blood draw (concentration of 35.7 ng/ml) and two withdrew after the second blood draw (first period mean concentrations of 9.6 and 25.4 ng/ml). Thus, 197 were followed for the first period, and 195 for the remainder of the study.

The study began on September 20, 2009, the date of the first blood draw, and ended on January 12, 2010, two days after the last blood draw. An interim analysis of the coded data 2-3 weeks after the second period of observation caused the investigators to decide to close the study after the fourth blood draw (the end of the third period).

### Acute infections evaluated

Four patients developed bacterial infections (streptococcal pharyngitis, two bacterial pneumonias, and diverticulitis). None of these infections was included in the analysis, as they were not acute viral respiratory tract infections.

Of the 103 clinical acute respiratory tract infections, 89 (86.4%) involved physician visits and 14 (13.6%) were documented by illness diary only. There were 103 acute viral infections diagnosed clinically in 84 patients during the study, including 62 afebrile respiratory tract infections, 8 ILI, and 33 laboratory confirmed infections. Of the 89 clinical viral infections in which virology studies were performed, 33 (37.1%) had positive results (7 2009 H1N1 influenza, 22 rhinovirus, 3 RSV, and 1 parainfluenza).

### 25-hydroxyvitamin D concentrations in the study group

**Figure 1:**
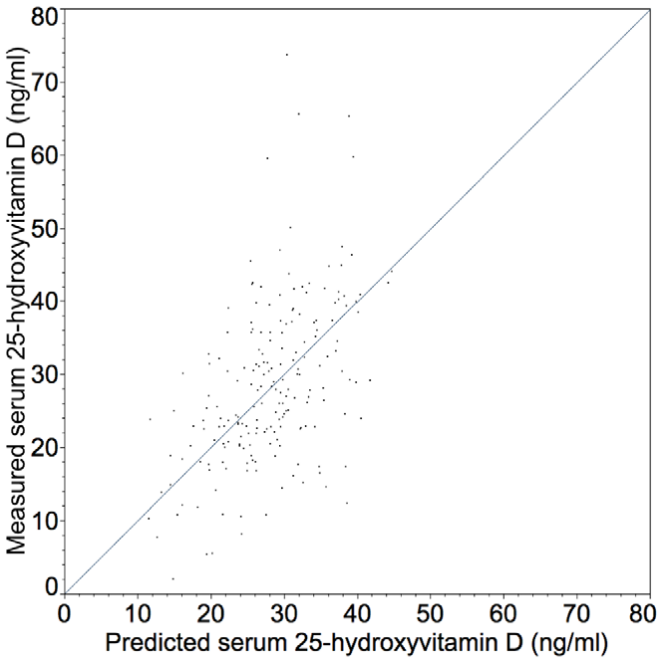
Predicted vs. observed serum 25-hydroxyvitamin D concentrations. Predicted from the equation [serum vitamin D ng/ml] = [427.4×(1+pigmentation grade)^0.5^+(dose IU/day) ^0.5^]/Body mass index, where body mass index = (weight lbs)(703)/height inches)^2^. See text for pigmentation grade. The observed concentrations were from the first observation period. Model parameters were SSE = 18755.12; DFE = 197; RMSE = 9.75. The error associated with the parameter estimate was 427.4 +/− 10.4 (SEM) (406.8–448.0) 95% CI.

The concentrations of 25-hydroxyvitamin D in the participants were consistent with what has been described in the literature, including the mean and range of values found; differences based on sex and skin pigmentation; and, seasonal decline (Table 1). The results of the initial non-linear model to examine the effects of population covariates on serum vitamin D concentration suggested that only skin pigmentation, vitamin D dose, and body mass index (BMI) were related to serum vitamin D concentrations measured in the first observation period (Figure 1).

**Table 1:** Serum 25-hydroxyvitamin D concentrations over the course of the study by gender and skin pigmentation.

	Mean serum 25-hydroxyvitamin D concentrations±standard deviation (range)							
	1^st^ blood draw (09/20/09–09/28/09)		2^nd^ blood draw (10/27/09–11/04/09)		3^rd^ blood draw (11/30/09–12/06/09)		4^th^ blood draw (12/30/09–01/10/10)	
**All participants**	N = 198	28.4±0.8 (2.0–73.7)	N = 197	27.0±0.8 (2.0–73.0)	N = 195	24.6±0.8 (2.0–56.2)	N = 195	25.6±0.8 (4.3–60.4)
**Female**	N = 113	29.4±1.1 (2.0–65.6)	N = 112	28.1±1.0 (2.0–59.5)	N = 111	26.5±1.1 (2.0–56.2)	N = 111	27.4±1.1 (4.3–60.4)
**Male**	N = 85	27.1±1.2 (8.2–73.7)	N = 85	25.5±1.1 (7.4–73.0)	N = 84	22.1±1.0 (5.6–47.2)	N = 84	23.1±1.0 (6.7–53.2)
**Light pigmentation**	N = 154	30.7±0.9 (5.4–73.7)	N = 153	29.0±0.9 (5.0–73.0)	N = 152	26.7±0.9 (2.0–56.2)	N = 152	27.7±0.9 (6.0–60.4)
**Intermediate pigmentation**	N = 32	22.0±1.1 (10.6–36.1)	N = 32	20.8±1.2 (8.6–39.6)	N = 31	18.3±1.2 (5.9–33.5)	N = 31	18.9±1.2 (6.7–34.1)
**Dark pigmentation**	N = 12	15.8±2.3 (2.0–30.1)	N = 12	17.4±3.0 (2.0-32.6)	N = 12	15.1±2.3 (2.0–31.3)	N = 12	16.8±2.4 (4.3–28.6)

### 25-hydroxyvitamin D concentrations and viral respiratory tract infections

**Figure 2:**
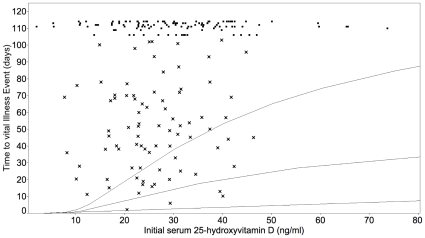
Length of time to viral infection related to initial serum concentration of 25-hydroxyvitamin D. Shown are the results of the pharmacodynamic model relating 25-hydroxyvitamin D to length of time before a viral respiratory tract infection. The model equation is Log(time to event) = b_0_+b_1_/(Maximal 25OH-Vit D–serum 25-OH vitamin D+1) calculated using a Weibull loss function. The parameters associated with the regression are as follows, given with 95% CI and P-values: b_0_ = 4.29 (3.50–4.96), <0.001; b_1_ = 38.33 (9.64–76.48), <0.001; Sigma = 0.79 (0.65–0.97), <0.001. Overall P-value for this model was <0.0023. Curves are fitted 0.1, 0.5 and 0.9 quantiles as a function of the regressor. The x points represent individuals who developed viral infections, and the other points represent individuals who did not develop infections and the three who were not followed until the end of the observation period. For the individuals who developed viral infections (x points) the mean age was 46.9 years; there were 47% men; 76.5% had light pigmentation, 18.8% intermediate pigmentation, 4.7% dark pigmentation; the mean initial 25-hydroxyvitamin D concentration was 26.12 ng/ml; and the mean vitamin D supplementation 292.5 IU. For the individuals who did not develop viral infections (the other points) the mean age was 47.0 years; there were 39.8% men; 78.8% had light pigmentation, 14.2% intermediate pigmentation, 7.0% dark pigmentation; the mean initial 25-hydroxyvitamin D concentration was 30.03 ng/ml; and the mean vitamin D supplementation 601.4 IU.

For each of the three periods of observation and for the entire study the partition analysis determined that a vitamin D concentration of 38 ng/ml best discriminated between groups that did or did not develop viral infections of the respiratory tract. This was consistent with the value of 38 ng/ml obtained from the pharmacodynamic concentration-response curve based on the initial 25-hydroxyvitamin D concentrations (Figure 2).

**Figure 3:**
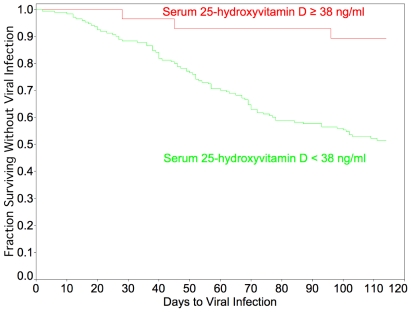
Survival without respiratory viral infection over course of study, stratified by serum 25-hydroxyvitamin D concentrations. Results of Cox proportional hazard analysis for time to viral infection, stratified by serum 25-hydroxyvitamin D concentrations. Parameters included 103 events (viral infections), 3 in the ≥38 ng/ml group and 100 in the <38 ng/ml group, and 114 censorings (15 without infection in the ≥38 ng/ml group, 99 in the <38 ng/ml group, and 3 lost to follow-up). The parameter estimate for 25-hydroxyvitamin D≥38 ng/ml was −0.66 (−1.36—0.17) 95% CI and the risk ratio 0.51 (0.25 to 0.84) 95% CI (p<0.0001).

The results of the Cox proportional hazard model are presented in Figure 3. A vitamin D concentration ≥38 ng/ml approximately halved the risk for development of an acute viral respiratory tract infection over the observation period (p<0.0001). The survival curve depicts events (viral infections). With respect to participants, 83.3% of the 18 who maintained vitamin D concentrations ≥38 ng/ml for the entire study survived uninfected, whereas only 55% (99/180) of the other participants survived without infection.

Analyses of the groups with concentrations ≥38 ng/ml and <38 ng/ml are delineated in Table 2. There were no differences due to ingestion of herbals, supplements, or vitamins other than D, or, due to receipt of seasonal influenza vaccine or 2009 H1N1 vaccine before the observation periods. The participants with the higher concentrations of vitamin D were more likely to have variables that were known from the literature and our non-linear model to be associated with higher vitamin D concentrations: lighter skin pigmentation and ingestion of vitamin D, the latter of which also explained the seemingly apparent difference in the high and low vitamin D groups based on gender. Only 24/85 (28.2%) of men were on vitamin D supplements, and only 4/85 (4.7%) took 1000 IU or more daily; the numbers for the women in the study were 55/113 (48.7%) and 26/113 (23.0%), respectively.

**Table 2:** Analysis^a^ of variables between groups with 25-hydroxyvitamin D concentrations of ≥38 ng/ml and <38 ng/ml.

	Period 1 (09/20/09–10/31/09)			Period 2 (11/01/09–11/30/09)			Period 3 (12/01/09–01/12/10)			Entire Study (09/20/09–1/12/10)		
**Mean 25-hydroxyvitamin D concentrations (ng/ml)**	≥38	< 38	P value	≥38	< 38	P value	≥38	< 38	P value	≥38	< 38	P value
**Total number in group**	32	166		23	175		24	174		18	180	
**Male/Female**	10/22	75/91	1	6/17	79/96	1	3/21	82/92	0.016	2/16	83/97	0.048
**Light pigmentation**	32	122	0.003	23	131	0.028	24	130	0.028	18	136	0.147
**Intermediate pigmentation**	0	32	0.031	0	32	0.296	0	32	0.166	0	32	0.487
**Dark pigmentation**	0	12	1	0	12	1	0	12	1	0	12	1
**Seasonal influenza vaccine before period**	2	7	1	14	123	1	14	123	1	11	126	1
**2009 H1N1 vaccine before period**	0	1	1	1	22	1	8	77	1	6	79	1
**Herbals/supplements**	9	34	1	9	33	1	8	34	1	6	36	1
**Vitamins without D**	1	7	1	1	6	1	1	6	1	1	6	1
**Vitamin D, any dose**	20	59	0.060	15	64	0.119	20	59	0.001	13	66	0.048
**Vitamin D≥1000 IU per day**	11	19	0.024	11	19	0.001	13	17	0.001	10	20	0.001

a Contingency tables calculating two-tailed p values using Fisher's exact test with Bonferroni correction for multiple testing were used.

To examine the burden of illness, the incidence of clinically diagnosed acute viral respiratory tract infections and percentages of days ill in participants with 25-hydroxyvitamin D concentrations ≥38 ng/ml and in those <38 ng/ml were determined (Table 3). For each observation period and for the entire study the incidence of viral respiratory infections and the percentage of days ill were significantly lower for the group with concentrations of 25-hydroxyvitamin D≥38 ng/ml. Indeed, for the group ≥38 ng/ml for the entire study period, the incidence of infection was 2.7 times lower (p = 0.0150). The percentage of days ill in the ≥38 ng/ml group compared to the <38 ng/ml group was 4.9 times lower; however, a precise multiple could not be determined, as proper analysis required knowledge of the exact range of illness duration in the higher group, and, as discussed below, that could not be determined due to the small number of infections (3) in that group. Obviously, unless the duration of illness in the group with the higher 25-hydroxyvitamin D concentrations was longer than in the other group, the 2.7 fold reduction in incidence would have to reduce the percentages of days ill by at least that factor in the higher group.

**Table 3:** Incidence of acute viral respiratory tract infections and number of days ill in groups with 25-hydroxyvitamin D concentrations of ≥38 ng/ml and <38 ng/ml.

Period of observation (dates)	Mean 25-hydroxy vitamin D	Number participants with level	Number ill participants (%) (95% CI)	P value^a^	Total days observed	Total days ill (%)
**Period 1 (09/20/09–10/31/09)**	≥38 ng/ml	32	1 (3.1%) (0.0–9.1)		1240	6 (0.48%)
	<38 ng/ml	166	30 (18.1%) (13.2–23.1)	p = 0.0137	6282	149 (2.4%)
**Period 2 (11/01/09–11/30/09)**	≥38 ng/ml	23	1 (4.3%) (0.0–12.5)		690	2 (0.29%)
	<38 ng/ml	175	37 (21.1%) (16.2–26.4)	p = 0.0237	5160	365 (7.1%)
**Period 3 (12/01/09–01/12/10)**	≥38 ng/ml	24	1 (4.2%) (0.0–12.0)		1032	8 (0.78%)
	<38 ng/ml	174	31 (17.8%) (13.2–22.7)	p = 0.0434	7353	263 (3.6%)
**Entire study (09/20/09–01/12/10)**	≥38 ng/ml	18	3 (16.7%) (4.2–33.3)		1994	16 (0.80%)
	<38 ng/ml	180	81 (45%) (38.9–51.1)	p = 0.0150	19763	777 (3.9%)

a Bootstrap p-values and confidence intervals (CI) over 100,000 trials.

### Duration of illness

For participants with 25-hydroxyvitamin D concentrations ≥38 ng/ml, the median duration of illness was 6 days (3 infections, range 2–8 days); for participants with concentrations <38 ng/ml, the median duration was also 6 days (100 infections, range 2–27 days). For influenza, the median durations for the high and low 25-hydroxyvitamin D groups were 2 days (1 infection with no antivirals given) and 9 days (6 infections, range 2–20 days, with antivirals in two participants), respectively. The number of infections in the participants with concentrations ≥38 ng/ml was too small to determine if these differences in illness duration were statistically significant.

### Infections with positive virology studies

The numbers of participants with mean concentrations ≥38 ng/ml were 32 for the first period, 23 for the second, and 24 for the third. Eighteen participants maintained concentrations ≥38 ng/ml for the entire study. Of the 18, 3 developed viral infections. Of the 180 other participants, 81 developed infections, and all of the 81 had mean concentrations of vitamin D<38 ng/ml for the entire study. Of the 18 participants with high vitamin D concentrations, there was one (5.5%) who developed a laboratory-confirmed viral infection. Of the other180 participants, there were 32 (17.8%) laboratory-confirmed cases. Due to the small number of infections in the ≥38 ng/ml group, the study had a low power to detect a difference in the incidence of laboratory-confirmed infections in the two 25-hydroxyvitamin D groups (p = 0.0978, by bootstrap analysis with >100,000 trials).

## Discussion

This study is the first prospective study that has correlated serum 25-hydroxyvitamin D concentrations with the incidence of viral respiratory infections in adult women and men. During 114 days of the fall and winter in a temperate zone a serum concentration of 25-hydroxyvitamin D of 38 ng/ml or higher was associated with a two-fold decrease (p<0.0001) in the risk of developing acute viral infections of the respiratory tract. The effect size was so large that it was demonstrated with confidence in a relatively small study. For three consecutive periods and for the whole study the incidences of infection for the group with 25-hydroxyvitamin D≥38 ng/ml were significantly lower (p values 0.0137, 0.0237, 0.0434, and 0.0150, respectively) than in the <38 ng/ml group.

Analysis based on the initial 25-hydroxyvitamin D concentrations revealed that the higher the concentration, the greater the reduction in the incidence of viral infections of the respiratory tract, with the effect plateauing at concentrations ≥38 ng/ml. The findings herein may explain the apparent lack of an effect of supplementation with vitamin D on reducing the incidence of viral infections of the respiratory tract in a study done by Li-Ng et al [37]. In that placebo-controlled interventional study a mean 25-hydroxyvitamin D concentration of 35 ng/ml was reached in the treatment group by the end of the study, with 27% still below 30 ng/ml. Had the concentrations reached ≥38 ng/ml, that study might have had the power to detect a difference in the treatment group.

The 25-hydroxyvitamin D concentrations observed in the present study were consistent with what is known about the pharmacology of vitamin D in humans. Importantly, increased melanin content in skin is known to block solar ultraviolet B radiation required for the first step of synthesis of the vitamin in the skin. The predicted vitamin D concentration ratio for individuals with intermediate and dark pigmentation compared to lightly pigmented individuals in this study was approximately 1/√2 and 1/√3, respectively, given no additional vitamin D intake and a similar body mass index. The 18 participants who were able to maintain 25-hydroxyvitamin D concentration ≥38 ng/ml for the entire study all had light skin pigmentation, and 72.2% were on vitamin D supplementation. The data suggested that there is a non-linear response in dose of vitamin D to serum concentration, which may be related to suggestions of increased metabolism of the vitamin in individuals with higher serum vitamin D concentrations [38]. The study also found an inverse relationship between body mass index and the serum concentration of the vitamin, consistent with a higher volume of distribution in individuals with a higher body mass index due to the high fat solubility of the vitamin.

There were a number of limitations in this study. First, the number of ill participants with concentrations ≥38 ng/ml was too small to determine whether once infected those participants had a statistically significant shorter duration of illness. A subsequent study will be required to determine the durations of illness caused by specific viruses examined in light of vitamin D concentrations.

It had been expected that there would be many cases of 2009 H1N1 influenza, but the number of cases in the geographic area of the study was relatively small, and a large percentage of the subjects had received the 2009 H1N1 vaccine by the second period of the study, the peak time for influenza in the study's location. The virology studies performed used multiplex DFA as the initial screen and PCR only for selected viruses. Testing did not include coronavirus or parainfluenza type 4. Nevertheless, the recovery rate of 37.1% in this study was similar to rates of 39% [39] and 32.1% [40] reported in the literature in studies of respiratory tract viral infections in healthy adults in which PCR tests for more viruses were done. However, in this study the small number of infections seen in the participants with concentrations of 25-hydroxyvitamin D≥38 ng/ml limited determination of the effect of 25-hydroxyvitamin D on the incidences of infection caused by specific viruses; further studies are needed.

The results of this study cannot be assumed to apply to other settings, but do call for other studies to determine if vitamin D concentrations have an effect on the following: (1) the incidence of acute viral respiratory tract infections in individuals under 18 years of age; (2) the incidence of viral infections affecting other organ systems and at other times of the year; (3) the course of bacterial, mycobacterial, and fungal infections; (4) the course of specific infections which can be worse in groups prone to lower concentrations of vitamin D, e.g., influenza in pregnant women and in the obese, tuberculosis in dark skinned individuals, and fungal infections in dark skinned patients. In addition, careful intervention studies would be required to evaluate any potential therapeutic effects of vitamin D in the course of established infections of any type.

Our data suggest that there is a threshold above which progressively higher concentrations of vitamin D do not result in further benefit in reducing the incidence of viral infections of the respiratory tract. Larger studies might be undertaken examining different ranges of vitamin D concentrations above 38 ng/ml and monitoring for any long-term beneficial or adverse effects, given the widespread presence of vitamin D receptors on so many cell types.

Importantly, this study demonstrated a beneficial effect of a serum concentration of vitamin D (38 ng/ml) only slightly higher than the concentration (30 ng/ml) currently believed to represent sufficiency [12]. It should be quite safe to supplement one's diet to achieve a concentration just above 38 ng/ml; such supplementation should be done with a diet sufficient in calcium. Our results need to be replicated in other populations, and since latitude, season, skin pigmentation, and body mass index all affect serum vitamin D concentrations, the cost-benefit of supplementation with or without laboratory testing needs to be determined.

It is estimated that 1 billion people worldwide have vitamin D concentrations under 30 ng/ml [12], and a much larger number would be expected to have concentrations under 38 ng/ml, as found in 81.3% of the participants for entire duration of this study. The average adult has 2–3 viral respiratory tract infections each year, resulting in countless physician visits, days lost from school and work, and enormous direct and indirect costs. A study in 2001 estimated that in the United States alone there were 500 million non-influenza viral respiratory tract infections each year, resulting in 189 million school days and 196 million workdays missed by the ill and their caregivers, with $40 billion dollars in costs [41].

### Conclusions

The data in this study suggests that supplementing with vitamin D to raise the concentrations in the general population to above 38 ng/ml could result in a significant health benefit by reducing the burden of illness from viral infections, at a minimum from viral infections of the respiratory tract in healthy adults living in temperate climates. Our findings may provide direction for and call for future interventional studies examining the efficacy of vitamin D supplementation in reducing the incidence and severity of specific viral infections, including influenza, in the general population and in specific subpopulations, such as pregnant women, dark skinned individuals, and the obese.
